# Tissue-specific responses to oxidative fuel source preference during heat stress in lactating dairy cows

**DOI:** 10.3168/jdsc.2024-0631

**Published:** 2024-09-18

**Authors:** M.D. Ellett, K.M. Daniels, M.D. Hanigan, B.A. Corl, G. Perez-Hernandez, C.L.M. Parsons, J.A. Melvin, D.W. Fausnacht, R.P. McMillan, L.H. Baumgard, R.P. Rhoads

**Affiliations:** 1 School of Animal Sciences, Virginia Tech, Blacksburg, VA 24061; 2 Department of Biology, Ferrum College, Ferrum, VA 24088; 3 Virginia Tech Metabolism Core, Virginia Tech, Blacksburg, VA 24061; 4 Department of Animal Science, Iowa State University, Ames, IA 50011

## Abstract

Prolonged exposure to high environmental temperatures results in an accumulated heat load that induces a heat stress (HS) response in dairy cattle. Heat stress compromises dairy farm profitability by reducing milk yield, altering milk composition, and hindering reproductive performance. The ability to alternate between carbohydrate and lipid sources for energy production is termed metabolic flexibility (Met Flex). The objective of this study was to evaluate the Met Flex of mammary, muscle, and liver tissue in lactating dairy cows under HS and thermoneutral (TN) conditions. Sixteen Holstein cows were assigned to 1 of 2 treatment groups: pair-feeding in TN conditions (PFTN) or HS conditions. All cows experienced a 4-d TN period with ad libitum intake followed by a 4-d treatment period. Heat stress cows were exposed to a temperature-humidity index (THI) ranging from 76 to 80 and the PFTN cows were exposed to a THI of 64. Milk production and health data were recorded twice daily. Semitendinosus biopsies were obtained on d 4 of each period and postmortem mammary and liver samples were obtained on d 4 of period 2. All tissue samples were assayed for Met Flex. Activity of mitochondrial (Mit) enzymes were assessed in skeletal muscle only. Four days of HS decreased milk yield, altered milk composition, and increased respiration rate and rectal temperatures. No differences in Met Flex were observed in mammary or liver tissue during period 2. However, HS, but not PFTN conditions, lowered Met Flex of skeletal muscle by 18.3% when compared with TN ad libitum feed intake conditions of period 1. No treatment differences were observed in skeletal muscle Mit enzyme activity indicating the decrease in Met Flex occurred independently of changes in Mit function. The reduction in Met Flex of skeletal muscle during HS may contribute to reduced milk yield and warrants further investigation.

## Highlights

• Four days of HS lowered metabolic flexibility in skeletal muscle.
• HS did not affect metabolic flexibility of mammary or liver tissue.
• HS reduced total milk and milk component yields.
• Respiration rates and rectal temperatures were elevated in response to HS.

## Graphical Abstract

**Figure:**
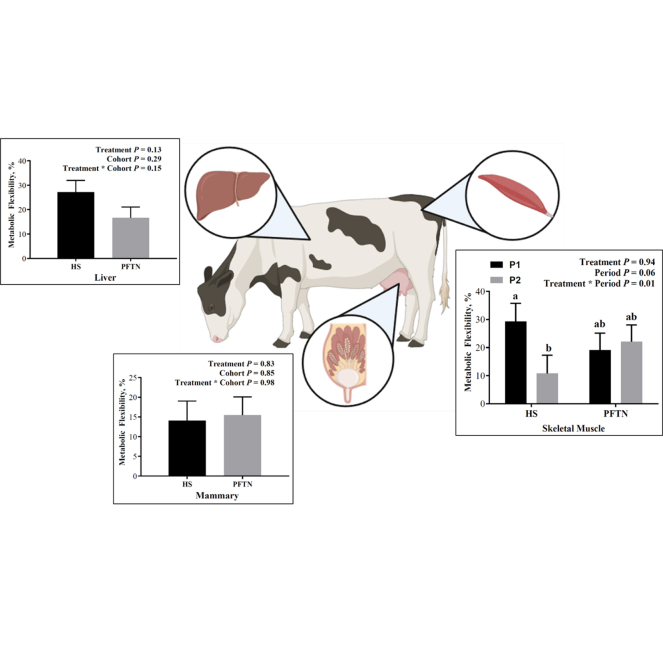
**Summary:** Heat stress (HS) lowers milk yield and alters milk component concentration independently of concurrent reduction in dry matter intake. The physiological mechanisms mediating this profit-lowering response to HS are not well defined. It is hypothesized that HS alters metabolism in a tissue-specific manner, altering glucosesparing mechanisms to increase dependency on glucose as a fuel source. The objective of this work was to elucidate the effects of HS on the metabolic flexibility (i.e., substrate adaptability) of muscle, liver, and mammary tissue in lactating dairy cattle. Heat stress reduced energy substrate adaptability in skeletal muscle but did not alter metabolic flexibility in mammary or liver tissue. Reduced metabolic flexibility in skeletal muscle was due to decreased lipid oxidation, resulting in an increased dependency on pyruvate oxidation. Altered metabolic flexibility may contribute to decreased milk yield and warrants further investigation.

Heat stress (**HS**) occurs when cattle accumulate heat loads that increase core body temperature higher than the upper critical limit of their thermoneutral (**TN**) zone. The accumulated heat load prompts a physiological response to decrease heat production and increase heat dissipation (Baumgard and Rhoads, 2013). Dairy cattle with high milk production are especially susceptible to HS due to increased whole-body metabolism to support milk synthesis (Coppock et al., 1982). It is commonly accepted that US dairy breeds elicit a HS response when temperature-humidity index (**THI**) value meets or exceeds 68 (Zimbelman et al., 2009). Signs of HS, such as decreased DMI, milk yield, and milk protein concentration and increased respiration rate and core body temperature are hallmark responses to HS (Chen et al., 2024). Compromised milk production and lower reproductive performance associated with HS results in decreased farm profitability, costing the US dairy industry more than $1.2 billion annually (Key and Sneeringer, 2014).

Pair-feeding studies have shown that reduced DMI can be responsible for 30% to 50% of the reduction in milk yield (Rhoads et al., 2009). Remaining milk yield reductions may be attributed to an altered ability to metabolize nutrients and a repartitioning of energy-yielding substrates. Heat-stressed lactating cows have elevated circulating insulin and BUN, and reduced concentrations of nonesterified fatty acids (**NEFA**), free AA, BHB, and glucose when compared with pair-fed TN counterparts (Gao et al., 2017). Conditions and diseases that alter macronutrient metabolism negatively affect animal health and performance (Haq et al., 2022). Milk yield is dependent on milk component yield, with lactose being the primary osmole influencing milk yield. Lactose synthesis is dependent on an adequate glucose supply, in part facilitated by glucose sparing mechanisms (Bauman and Currie, 1980).

The ability to alter metabolism in response to available substrates is termed metabolic flexibility (**Met Flex**) and is an important indicator of metabolic health (Goodpaster and Sparks, 2017). In humans, reduced Met Flex is linked to diabetes (Galgani et al., 2008) and cancer (Dart, 2016). Reduced liver and skeletal muscle Met Flex is associated with reduced milk production in LPS-infused lactating dairy cattle (Opgenorth et al., 2024) and reduced skeletal muscle Met Flex pig performance during HS (Zhao et al., 2018). Evidence of altered whole-body insulin signaling, glycemia, and lipid metabolism due to HS in dairy cattle (Fontoura et al., 2022) suggests that HS dairy cattle may also experience reduced Met Flex. The objective was to examine the regulation of fuel substrates in skeletal muscle, mammary, and liver tissue of lactating dairy cattle under HS conditions by assessing Met Flex. Due to the mammary gland being in a lipogenic state, and a lack of LPS-mediated immune activation reported in the companion paper (Ellett et al., 2024), it was hypothesized that HS would alter Met Flex in skeletal muscle without altering Met Flex in the mammary and liver tissue.

All procedures were approved by the Virginia Tech Institutional Animal Care and Use Committee (protocol #19–247). Information pertaining to experimental design, animal treatments, husbandry, milk yield, and component data, and respiration and rectal temperature monitoring is published elsewhere (Ellett et al., 2024). Briefly, 16 multiparous Holstein cows (632 ± 46 kg BW; mean ± SD) approximately 100 DIM were transported to the Metabolic Research Laboratory at the Virginia Tech Dairy Complex (Blacksburg, VA). The overall experiment was performed using 2 cohorts with 8 cows per cohort. Upon arrival, all cows were placed in 1 of 2 temperature-controlled rooms for 4 d of acclimation to tiestall housing, milking in place, and a common TMR. Cows were milked twice daily, had unlimited access to water, and were fed a TMR balanced for milk yield and components twice daily.

Temperature-humidity index was calculated using the equation from Davis et al. (2003): THI = 0.8 × T + [RH × (T − 14.4)] + 46.4, where T = mean hourly ambient temperature in °C and RH = mean hourly relative humidity divided by 100. Cows were under TN conditions during the acclimation period (THI = 64). Cows were enrolled into either a HS or pair-feeding in TN conditions (**PFTN**) treatment group on the last day of the acclimation period. One cow was removed due to a non-treatment-related shoulder injury, and the animal's data were removed from all datasets; final animal numbers were 7 for the HS group and 8 for PFTN. The acclimation period was followed by a 4-d TN period (**P1**) and then a 4-d treatment period (**P2**). In P1, all cows were housed under TN conditions and had ad libitum access to TMR for 4 d. In P2, HS cows were exposed to cyclical HS (THI = 74–80) for 4 d. In P2 for PFTN, THI remained at 64 and the previous day's average DMI for HS room was applied to the room.

On h 95 ± 1 h (d 4) of both P1 and P2, ∼1 h after morning milking, semitendinosus muscle was collected via a core muscle biopsy as described previously (Xie et al., 2016). Muscle tissue (2–3 g) was placed in metabolic buffer (0.25 *M* sucrose, 1 m*M* EDTA, 0.01 *M* Tris·HCl, and 2 m*M* ATP) and frozen at −80°C for analyses. The biopsy obtained on d 4 of P2 was collected on the contralateral side in the same location as the biopsy obtained on d 4 of P1. At the end of P2, all cows were slaughtered for an aligning, but separate hypothesis, via penetrating captive bolt and exsanguination. Mammary tissue (∼2.5 g from the mid-section of the right, rear gland) and liver tissue (∼2.5 g) were collected and snap-frozen in liquid nitrogen within 15 min of slaughter. Frozen mammary and liver samples were stored at −80°C for Met Flex analysis.

Frozen muscle samples were sent to The Metabolism Core at Virginia Tech (Blacksburg, VA) to spectrophotometrically assess enzyme activities of cytochrome C oxidase (**CcO**; EC 7.1.1.9), citrate synthase (**CS**; EC 2.3.3.16), and β-hydroxyacyl-CoA dehydrogenase (**BHAD**; EC 1.1.1.211) as described by Heilbronn et al. (2005).

Muscle, mammary, and liver samples were assessed for Met Flex by comparing pyruvate ([1–^14^C] pyruvate) oxidation in the presence or absence of 100 μ*M* palmitic acid as described (Zhao et al., 2018). When a reduction (% decrease) in pyruvate oxidation in the presence of palmitic acid is observed (presented in nmol/mg of protein per min), it suggests a greater ability to switch between pyruvate and palmitic acid and thus a greater Met Flex (Tarpey et al., 2017).

All data were analyzed using SAS, version 9.4 (SAS Institute Inc., Cary, NC). Before analysis data were tested for normality with the Shapiro-Wilks test using the UNIVARIATE procedure. All data were analyzed using the GLIMMIX procedure. Treatment (HS or PFTN), cohort (1 or 2), period (1 or 2), and their interactions were fixed effects in the model; cow nested within treatment and cohort was the random term. Period was not included in the model for mammary and liver data because mammary and liver samples were not collected during P1. Least squares means with SE are reported, and fixed effects were considered significant when Bonferroni-adjusted *P* < 0.05 and considered a tendency when Bonferroni-adjusted *P* < 0.1. If the treatment × period interaction had *P* < 0.1, the SLICE procedure was used to make pairwise comparisons of the effect of one factor within the other.

Four days after the imposition of treatment (HS or PFTN), milk yield, milk protein, milk fat, and other solids yields were reduced (period *P* < 0.01). Production data results and discussion can be found in Ellett et al. (2024). Four days after exposure to HS conditions lowered milk yield, protein, and other solids yields, increased core body temperature, and increased respiration rate 4 d after exposure to HS (Table 1). Milk fat yield tended to differ by treatment × period when period was in the model. The slice test showed that HS lowered milk fat yield more than pair-feeding (*P* < 0.01; 0.87 vs. 1.05 kg/d for HS and PFTN, respectively).

**Table 1:** The effect of heat stress (HS) and pair-feeding in thermoneutral conditions (PFTN) on milk production and physiology

Parameter	Period 1^1^		Period 2^2^		SEM^4^	*P-*value^3^						
	HS	PFTN	HS	PFTN		TRT	PER	Cohort	TRT × PER	TRT × Cohort	PER × Cohort	TRT × PER × Cohort
Milk yield, kg/d	43.2^a^	41.7^a^	33.2^b^	37.5^c^	2.0	0.81	<0.01	0.07	0.01	0.76	0.19	0.55
Milk component												
Fat, kg/d	1.65^a^	1.53^a^	1.24^b^	1.42^b^	0.1	0.99	<0.01	0.07	0.07	0.21	0.54	0.54
Protein, kg/d	1.24^a^	1.20^a^	0.87^b^	1.05^c^	0.1	0.52	<0.01	0.39	<0.01	0.69	0.78	0.95
Other solids, kg/d	2.54^a^	2.46^a^	1.96^b^	2.17^c^	0.1	0.83	<0.01	0.08	0.01	0.92	0.19	0.73
Respiration rate, breaths/min	43^a^	41^a^	80^b^	35^c^	2.0	<0.01	<0.01	0.01	<0.01	0.70	0.28	0.04
Rectal temperature, °C	38.30^a^	38.40^a^	40.00^b^	38.06^a^	0.1	<0.01	<0.01	0.65	<0.01	0.98	0.62	0.62

a–c Differing superscripts within a row indicate a significant difference (*P* ≤ 0.05).

1 During period 1, all cows in both treatments were treated the same. Cows were housed in thermoneutral conditions (temperature-humidity index of 64) and were fed ad libitum. Values listed are the treatment (TRT) by period (PER) LSM.

2 During period 2, the cows on the HS treatment group were exposed to cyclical HS conditions (temperature-humidity index ranging from 74–80). The PFTN cows were exposed to thermoneutral conditions (temperature-humidity index of 64), and their intake was reduced to match the intake of the HS cow. Values listed are the TRT by PER LSM.

3 *P*-values listed represent the fixed effects of TRT, PER, cohort, and their subsequent interactions.

4 The SEM calculation represents the highest SEM for TRT by PER and contains 7 cows.

Cytochrome C oxidase activity in the semitendinosus muscle differed by cohort (*P* = 0.01), tended to differ by cohort by period (*P* = 0.07), and differed by treatment by cohort by period (*P* < 0.01). Neither CS nor BHAD activities were affected by any model term (Figure 1). Skeletal muscle pyruvate oxidation in media lacking palmitic acid did not depend on treatment nor cohort but, skeletal muscle pyruvate oxidation in the presence of palmitic acid differed by period by treatment (*P* < 0.01). Skeletal muscle Met Flex tended to be higher in P1 than P2 (*P* = 0.06), differed by cohort (*P* = 0.05), and differed by treatment by period (*P* = 0.01; Figure 1). The slice test showed that the HS treatment group had a lower muscle Met Flex in P2 when compared with the TN, ad libitum feed intake conditions of P1 (*P* < 0.01; 10.8% vs. 29.1% for P2 and P1, respectively), but not less than pair-feeding (*P* = 0.22; 10.8% vs. 22.1% for HS and PFTN, respectively).

**Figure 1:**
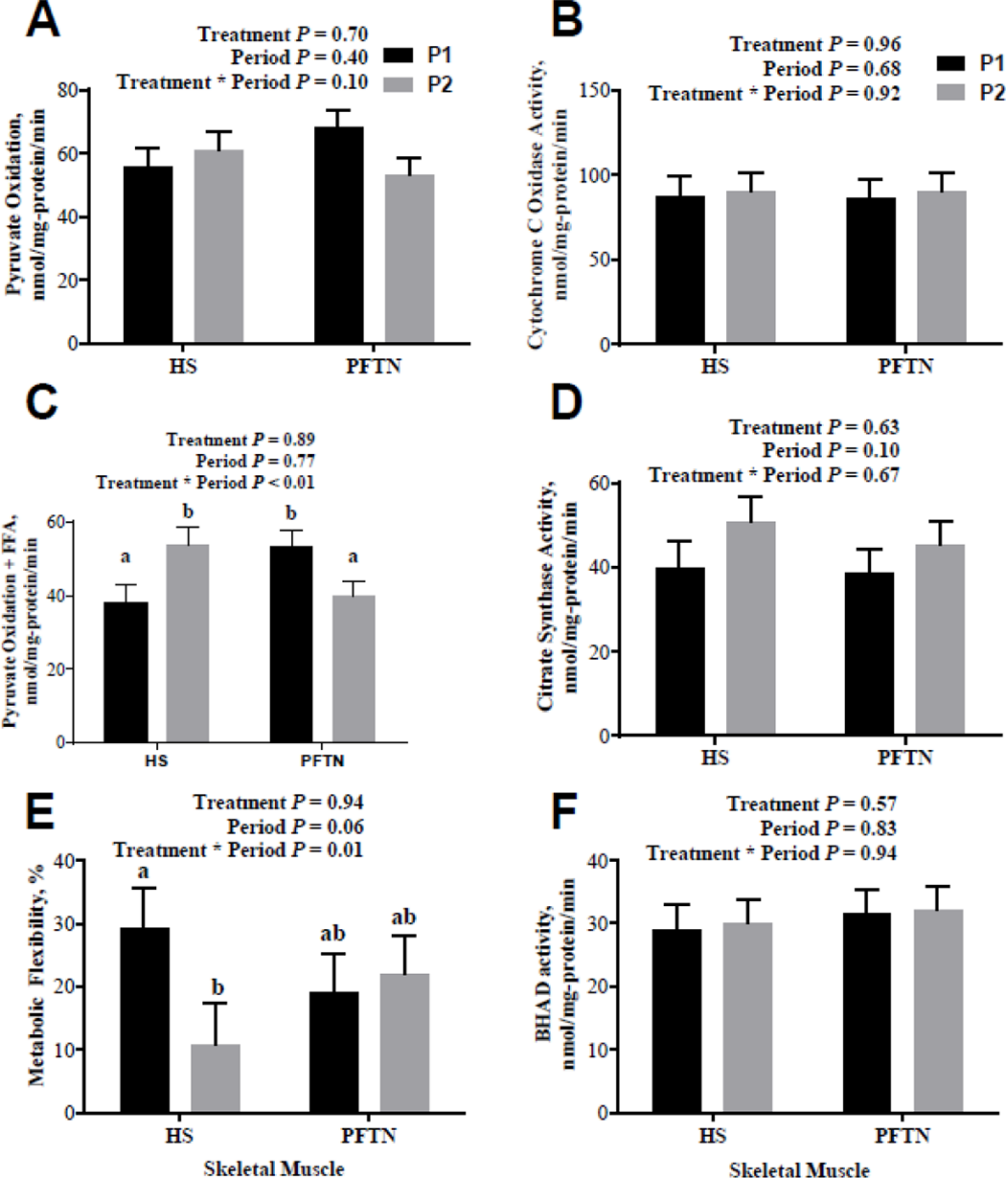
Effects of exposure to heat stress (HS) conditions or pair-feeding in thermoneutral conditions (PFTN) on (A) skeletal muscle pyruvate oxidation, (B) cytochrome C oxidase activity, (C) pyruvate oxidation in the presence of palmitic acid, (D) citrate synthase activity, (E) metabolic flexibility, and (F) BHAD activity. Differing letters denote a significant treatment by period difference of *P* < 0.05. Data are represented as treatment by period LSM ± SEM. The baseline thermoneutral data are represented by P1, and the treatment period (HS or PFTN) is represented in P2.

Mammary pyruvate oxidation in the presence of palmitic acid was lower in the HS treatment group than in the PFTN (*P* = 0.02; Figure 2). No differences in pyruvate oxidation in the absence of palmitic acid, and Met Flex were observed in the mammary tissue. Pyruvate oxidation in the presence, or absence, of palmitic acid and Met Flex did not differ in liver tissue (Figure 2).

**Figure 2:**
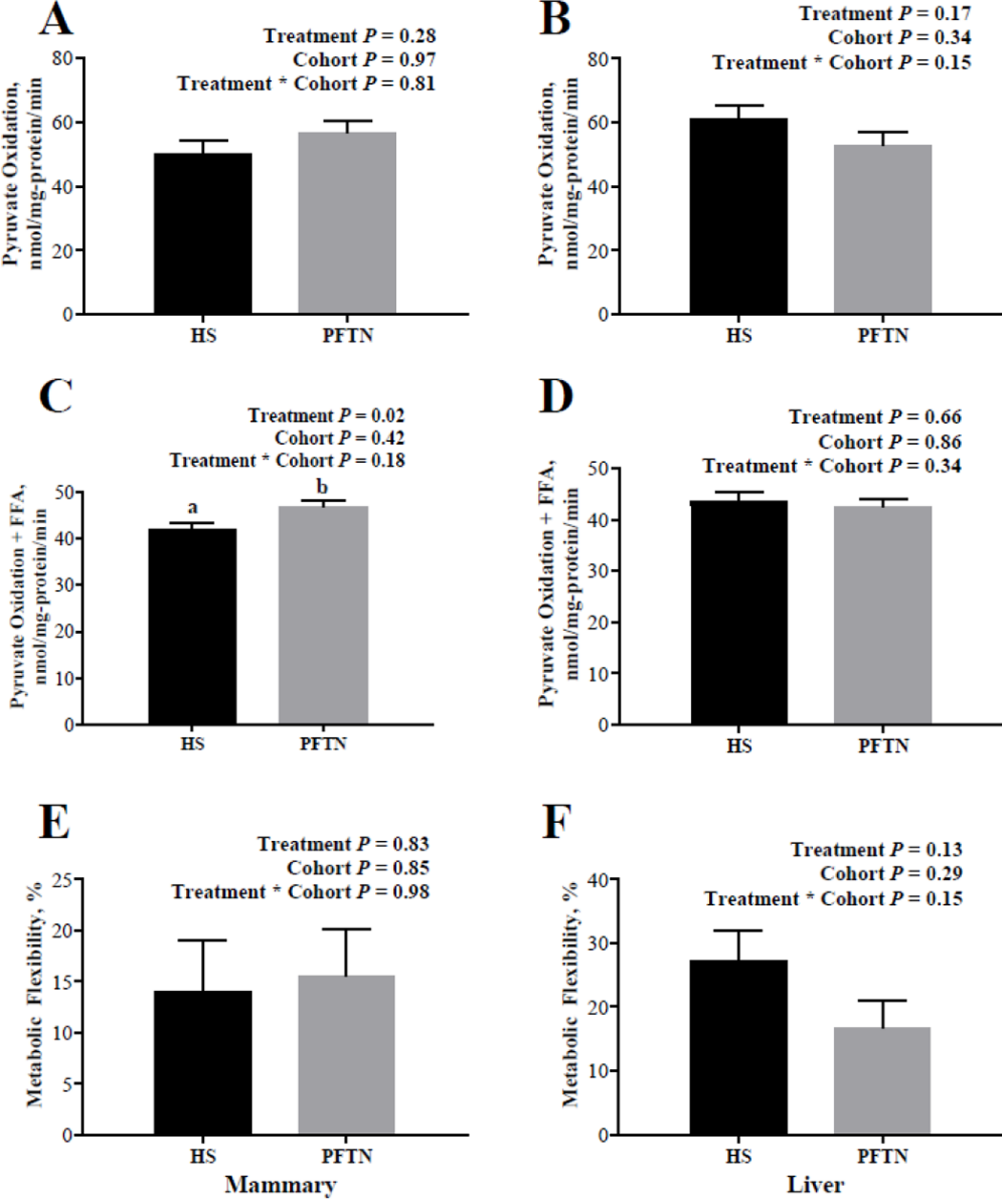
Effects of exposure to heat stress (HS) conditions or pair-feeding in thermoneutral conditions (PFTN) on (A) mammary pyruvate oxidation, (B) liver pyruvate oxidation, (C) mammary pyruvate oxidation in the presence of palmitic acid, (D) liver pyruvate oxidation in the presence of palmitic acid, (E) mammary metabolic flexibility, and (F) liver metabolic flexibility. Differing letters denote a significant treatment by period difference of *P* < 0.05. Data are represented as treatment LSM ± SEM.

In support of our hypothesis, findings from this study demonstrate that HS decreased Met Flex in the skeletal muscle of lactating dairy cows, consistent with previous findings in growing pigs (Zhao et al., 2018; Kroscher et al., 2022). This suggests a conserved phenotype that may be observed across multiple livestock species. Skeletal muscle appears to be more susceptible to losses in Met Flex than other tissues; liver and mammary tissue from the cows did not sustain significant reductions in Met Flex due to HS. Differences may have been observed in liver and mammary tissue if samples were collected in P1. Changes in muscle metabolism during HS can conceivably influence substrate availability throughout the entire body, particularly in growing animals. In support of this, in heat-stressed growing pigs, a loss of muscle Met Flex prevents skeletal muscle from oxidizing lipids for energy, causing an increase in the uptake and oxidation of carbohydrates (Zhao et al., 2018). Changes in substrate preference and reductions in lipid oxidation due to HS compromise total energy yield in meat and milk producing animals and reduce growth (Mitlöhner et al., 2001). In dairy cattle, increased glucose uptake by muscle tissue during HS (Bernabucci et al., 2010) would remove glucose from the circulating pool. This would, in turn, limit the quantity of glucose available for uptake by mammary epithelial cell (**MEC**), potentially resulting in decreased lactose yields (Baumgard and Rhoads, 2013). The companion article, Ellett et al. (2024), reports lower blood glucose in the HS treatment group when compared with the PFTN cows (treatment, *P* = 0.05). Unfortunately, milk lactose was not directly quantified, but milk other solids, which includes lactose and minerals, were measured. The HS cows had lower milk other solids when compared with PFTN.

Ruminant MEC, in contrast to those of nonruminants, primarily uses acetate as the main energy-yielding (i.e., oxidative) substrate due to its high circulating concentration relative to glucose. Lactating bovine MEC are lipogenic and primarily use acetate and BHB, not glucose, for de novo fatty acid synthesis (Collier, 1985). Being in a lipogenic state makes it unlikely that fatty acids are oxidized as a fuel source in MEC of lactating cows, thus making MEC relatively metabolic inflexible. The difference observed in pyruvate oxidation in the presence of palmitic acid may be due to the composition of the mammary tissue used in this experiment. Using samples collected from the same set of cows, Perez-Hernandez et al. (2024), report a greater number of alveoli per unit area in response to HS; different concentrations of myoepithelial cells, other connective tissue cells, or both, may explain the difference in pyruvate oxidation observed in the present study.

Lactate is a gluconeogenic substrate that contributes to the pool of available glucose (Aschenbach et al., 2010). Despite increased blood flow during HS, skeletal muscle upregulates lactate dehydrogenase resulting in an increased conversion of pyruvate to lactate (Baumgard and Rhoads, 2013). Lactate is then secreted into the blood, absorbed by the liver, and converted into glucose; a process known as the Cori cycle (Cori, 1940). Heat stress is also known to increase lactate concentrations in the rumen, which presumably increases lactate absorption and contributes to the elevated concentration of plasma lactate (Zhao et al., 2019). Aside from use in gluconeogenesis, some tissues may directly convert lactate to pyruvate and then oxidize it in the tricarboxylic acid cycle (Brooks, 2007). Lactate also plays a multifaceted role in regulating lipid metabolism. It functions as an antilipolytic hormone that reduces the mobilization of NEFA from adipose tissue, thus reducing the NEFA pool available for oxidation (Brooks, 2009). Lactate metabolism is also known to reduce the entry of NEFA into the mitochondria (**Mit)** by blunting the expression of carnitine palmitoyl transferase 1 (Brooks, 2009). Considered together, lactate metabolism lowers the available NEFA and the ability of NEFA to be transported into the Mit. The combination of increased reliance on AA and lactate as gluconeogenic precursors, and blunted glucose sparing mechanisms can lead to compromised milk yield and protein content during HS (Baumgard and Rhoads, 2013).

It is difficult to discern if the metabolic adaptations to HS are an intentional programmed response or a result of physiological damage at the cellular level. During HS, cells can incur excess oxidative damage that can reduce the function and number of Mit (Slimen et al., 2014). In addition, lipid oxidation occurs primarily in Mit, and a loss of Mit function impairs lipid oxidation and Met Flex (Slimen et al., 2014). However, we detected a robust decrease in Met Flex due to HS, yet Mit enzyme function was unaffected by treatment. No observable difference in BHAD activity indicates the ability to oxidize fats was not affected by HS and that palmitic acid oxidation was likely limited due to the reduced transport into the Mit. Citrate synthase activity, which was not affected by HS in this study, is used as an indicator of Mit number (Larsen et al., 2012). Cohort differences observed in CcO (cohort 1 LSM 68.4 vs. cohort 2 LSM 108.8 nmol/mg of protein per min) are result of animal variation, which may be attributed to the source herd or a result of an acclimation to a HS inducing environment. Both cohorts were acquired from different farms and cohort 1 was enrolled on the trial in May, whereas cohort 2 was enrolled at the end of July. Exposure to HS inducing environments over the summer months may have conditioned cows enrolled in cohort 2 to be more resilient to HS. Animals that have adapted to prolonged HS have increased CcO protein expression in skeletal muscle (Tamura et al., 2014; Crilly et al., 2016), which may explain higher CcO activity values observed in cohort 2.

Changes in Met Flex presenting without changes in Mit function indicate altered substrate preference mechanistically precedes Mit dysfunction. In the cows from this study, greater reductions in skeletal muscle Met Flex were observed in conjunction with decreased milk production and increased rectal temperature. These findings warrant further exploration into methods to ameliorate this reduction in Met Flex as a method to mitigate HS related production losses.

Decreased productivity in response to HS is a yearly billion-dollar burden for the dairy industry. Providing insights into the organ-level metabolic responses to HS will help elucidate the myriad of factors that result in a reduction in milk yield. The objective of this study was to evaluate the influence of HS on skeletal muscle, liver, and mammary fuel source metabolism by assessing Met Flex. Heat stress decreased Met Flex in skeletal muscle, indicating suppressed use of lipids as a fuel source during HS. The lack of a treatment difference in Mit enzyme activity in skeletal muscle indicates that the reduced utilization of lipid as a fuel source presumably occurred in the absence of altered Mit function. The reduction in Met Flex during HS may contribute to reduced milk production.
